# Left atrial size predicts outcome in severe but asymptomatic mitral regurgitation

**DOI:** 10.1038/s41598-023-31163-0

**Published:** 2023-03-08

**Authors:** Robert Zilberszac, Andreas Gleiss, Massimo Massetti, Wilfried Wisser, Thomas Binder, Harald Gabriel, Raphael Rosenhek

**Affiliations:** 1 Department of Cardiology, Vienna General Hospital, Medical University of Vienna, Waehringer Guertel 18-20, 1090 Vienna, Austria; 2 Center for Medical Statistics, Informatics, and Intelligent Systems, Medical University of Vienna, Vienna, Austria; 3 Institute of Cardiology, Catholic University of Sacred Heart, Rome, Italy; 4 Department of Cardiac Surgery, Medical University of Vienna, Vienna, Austria

**Keywords:** Cardiology, Medical research

## Abstract

Patients with severe asymptomatic primary mitral regurgitation (MR) can be safely managed with an active surveillance strategy. Left atrial (LA) size is affected by MR severity, left ventricular function and is also associated with the risk of atrial fibrillation and may be an integrative parameter for risk stratification. The present study sought to determine the predictive value of LA size in a large series of asymptomatic patients with severe MR. 280 consecutive patients (88 female, median age 58 years) with severe primary MR and no guideline-based indications for surgery were included in a follow-up program until criteria for mitral surgery were reached. Event-free survival was determined and potential predictors of outcome were assessed. Survival free of any indication for surgery was 78% at 2 years, 52% at 6 years, 35% at 10 years and 19% at 15 years, respectively. Left atrial (LA) diameter was the strongest independent echocardiographic predictor of event-free survival with incremental predictive value for the thresholds of 50, 60 and 70 mm, respectively. In a multivariable analysis that encompassed age at baseline, previous history of atrial fibrillation, left ventricular end systolic diameter), LA diameter, sPAP > 50 mmHg and year of inclusion, LA diameter was the strongest independent echocardiographic predictor of event-free survival (adjusted HR = 1.039, p < 0.001). LA size is a simple and reproducible predictor of outcome in asymptomatic severe primary MR. In particular, it may help to identify patients who may benefit from early elective valve surgery in heart valve centers of excellence.

## Introduction

Primary mitral regurgitation (MR) is common and its prevalence is expected to increase in an ageing society^1^. While many patients are asymptomatic and free from surgical triggers upon diagnosis, they might develop symptoms, left ventricular (LV) dysfunction, atrial fibrillation (AF) and pulmonary hypertension (PH) and those findings warrant surgery according to current guideline recommendations^2,3^. These recommendations however, are mainly derived from studies that have defined preoperative determinants of long-term postoperative outcome and studies describing predictors of outcome that permit risk stratification in asymptomatic patients are scarce. While in these patients, active surveillance until guideline-based indications for surgery are reached is associated with favorable outcome^4^, the identification of predictors of outcome that could help in the selection of patients who are likely to benefit from early surgery is nonetheless highly desirable, so as to further optimize the management of these patients. In this regard, quantitative determinants of regurgitation severity have been reported to predict survival in asymptomatic patients^5^, but several technical caveats apply when using these parameters^6,7^. The present study sought to define echocardiographic predictors of outcome in a large cohort of asymptomatic patients with severe primary MR.

## Methods

### Patient population

All consecutive patients with severe primary mitral regurgitation, who were studied in our outpatient heart valve clinic (HVC) between 1997 and 2015 were included in the study when they had no clinical or echocardiographic indications for surgery. Exclusion criteria were previous cardiac surgery or additional hemodynamically significant valve lesions (moderate or severe) except for tricuspid regurgitation (TR). According to these criteria, 280 consecutive patients (88 females) were identified. These patients form the study population for a previous paper^4^. The study protocol complies with the Declaration of Helsinki and was approved by the ethics committee of the Medical University of Vienna and was exempted from informed consent requirements owing to its observational study design**.**

### Clinical and echocardiographic data

At baseline, a comprehensive clinical assessment, including medical history, current medication, physical examination, electrocardiogram, blood tests, and transthoracic echocardiography, was performed. All patients underwent a comprehensive echocardiographic examination by an experienced echocardiographer. Quantification of MR severity was based on an integrated approach as recommended^7,8^; valve morphology, cavity sizes, and LV function were assessed. Left atrial (LA) diameters are derived from apical four chamber views measured at the end of systole, the diameter being measured parallel to the interatrial septum from the plane of the mitral annulus to the roof of the atrium. A LA diameter < 51 mm was considered normal, 51–60 mm mildly enlarged, 61–70 mm moderately enlarged and ≥ 70 mm severely enlarged.

### Follow-up

Patients were followed prospectively after their initial visit, based on a standardized HVC follow-up program described previously^4^. For the evaluation of outcome, events were defined as development of any criteria that indicated surgery or cardiac death related to MR. For the assessment of survival, the national mortality registry was queried and additional follow-up information was obtained from telephone interviews with the patients, their relatives, and their physicians.

### Statistical analysis

Categorical variables are described by counts and percentages. Continuous variables are described by medians and quartiles due to non-symmetric distributions for most of them. The distributions of event-free survival and overall survival are estimated by Kaplan–Meier curves and compared between categories using the log-rank test. Simple and multivariable Cox regression models are used to assess the effect of various predictors on survival. Hazard ratios (HR) are given with 95% confidence intervals (CI). Each predictor was checked for potential non-linear effects by adding and testing quadratic and cubic terms. Multiple models adjust for potential confounders of the effect of predictors of interest on survival, including year of inclusion to account for possible changes of patient cohort composition over the long recruitment period. The proportion of explained variation (PEV) is reported to quantify the importance of relevant predictors for individual prediction of survival^9^. While marginal PEVs give the importance of each predictor on its own, partial PEVs give the variation in survival that is additionally explained when adding the considered predictor to a list of other predictors.

The same analyses are applied to the cohort starting from 2005 to compare the predictive importance of LA diameter and LA volume.

All analyses were performed in SAS 9.4 (SAS Institute Inc., 2016). P-values are below 0.05 are regarded to indicate statistical significance.

## Results

The baseline patient characteristics are given in Table 1.

**Table 1:** Baseline patient characteristics.

Variable	Value
Gender (female), n (%)	88 (31%)
Age, years (quartiles)	58 (45–68)
Body mass index, kg/m^2^ (quartiles)	24 (22–27)
Coronary artery disease, n (%)	30 (11%)
Hypertension, n (%)	117 (42%)
Hypercholesterolemia, n (%)	54 (19%)
Diabetes mellitus, n (%)	13 (5%)
Statin, n (%)	37 (13%)
ACE inhibitor or angiotensin II receptor blocker, n (%)	97 (35%)
Betablocker, n (%)	71 (25%)
Acetylsalicylic acid**,** n (%)	51 (18%)
Previous bout of atrial fibrillation	37 (13%)
Systolic pulmonary artery pressure, mmHg (quartiles)	36 (30–45)
Right ventricular end-diastolic diameter, mm (quartiles)	32 (29–35)
Left ventricular end-systolic diameter, mm (quartiles)	33 (30–36)
Left ventricular end-diastolic diameter, mm (quartiles)	54 (50–58)
Left ventricular end-diastolic volume, ml (quartiles)	136 (108–166)
Indexed left ventricular end-diastolic volume, ml/m^2^ (quartiles)	71 (59–84)
Left ventricular ejection fraction, % (quartiles)	65 (62–68)
Left atrial diameter, mm (quartiles)	58 (52–62)
Left atrial volume, ml (quartiles)	99 (80–131)
Indexed left atrial volume, ml/m^2^ (quartiles)	53 (42–68)

Median (quartiles) and count (%).

### Event-free survival

As described previously^4^, during a median potential follow-up of 93.4 (quartiles: 55.3 to 152.9) months, 161 patients developed criteria warrating surgery and 13 patients died. Event-free survival rates at 2, 6, 10 and 15 years were 78.0% (95% CI: 73.2–83.2%), 52.2% (95% CI: 46.3–59.0%), 35.5% (95% CI: 29.3–43.1%), and 18.7% (95% CI: 12.3–28.5%), respectively.

Indications for surgery included the following: symptoms (n = 115), mild LV dysfunction (n = 11), LV dilatation (n = 7), de novo atrial fibrillation (n = 10), PH (n = 9), endocarditis (n = 7), before major noncardiac surgery (n = 1), and patient’s request(n = 1).

### Predictors of event-free survival

LA diameter was a highly significant independent predictor of subsequent events and provided incremental prognostic value (PEV = 6.2%).

Event-free survival rates for patients with an LA diameter ≤ 50 mm were 88.7% (75.0–95.1%) at 2 years, 68.5% (50.8–80.9%) at 6 years and 60.1% (40.9–74.8%) at 10 years.

Patients with an LA diameter of 51-60 mm had event-free survival rates of 80.8% (72.9–86.6%) at 2 years, 57.6% (48.0–66.0%) at 6 years and 41.6% (30.9–51.9%) at 10 years.

Patients with an LA diameter of 61–70 mm had event-free survival rates of 77.6% (66.0–85.6%) at 2 years, 46.5% (34.1–58.0%) at 6 years and 21.7% (11.9–33.4%) at 10 years.

**Figure 1:**
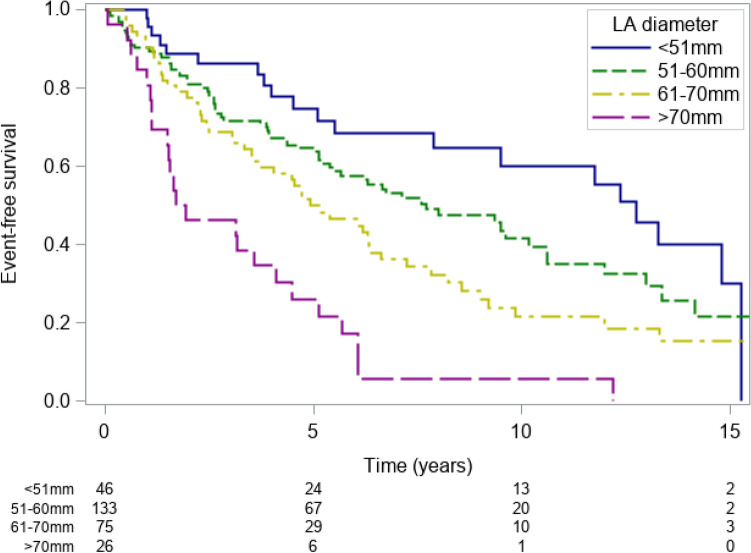
Kaplan–Meier event-free survival estimates for patients with LA diameter < 50 mm (blue line), 50-60 mm (green line), 60-70 mm (yellow line) and ≥ 70 mm (violet line). *LA* left atrium.

Patients with an LA diameter > 70 mm had event-free survival rates of 46.2% (26.6–63.6%) at 2 years, 17.3% (5.6–34.4%) at 6 years and 5.8% (0.5–22.2%) at 10 years (p < 0.001, Fig. 1).

**Figure 2:**
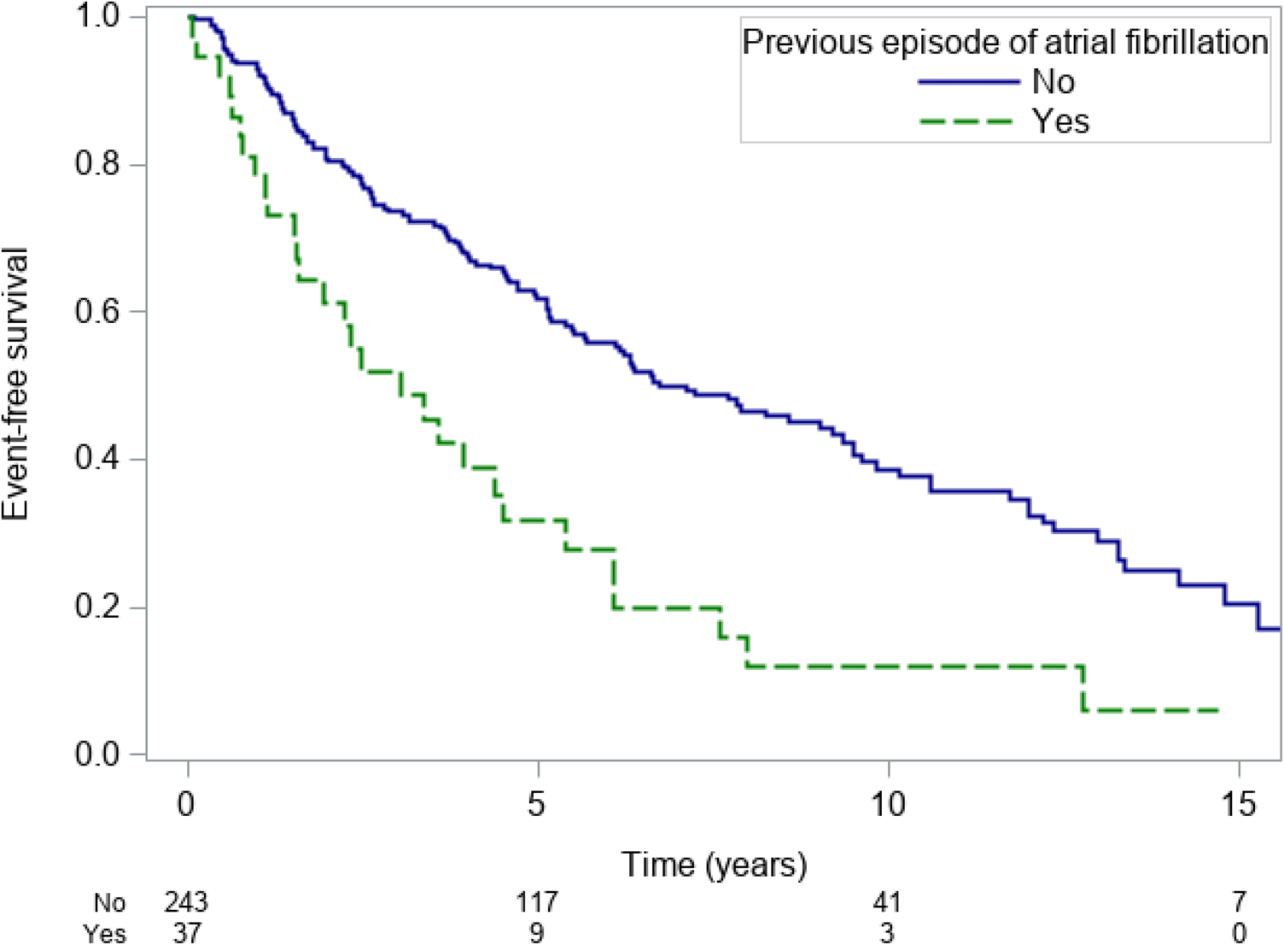
Kaplan–Meier event-free survival estimates for patients with previous AF (green line) and no previous AF (blue line). *AF* atrial fibrillation.

In addition, only low prognostic value was provided by a previous episode of AF with respective event-free survival rates of 61.1% (43.2–74.9%), 27.7% (13.4–44.1%) and 11.9% (3.1–26.9%) at 2,6 and 10 years respectively for patients with a previous episode of AF and event-free survival rates of 80.4% (74.7–84.9%), 55.9% (48.9–62.3%) and 38.7% (31.1–46.1%) at 2,6 and 10 years respectively for patients without a previous history of AF (p < 0.001; PEV = 3.0%, Fig. 2).

**Figure 3:**
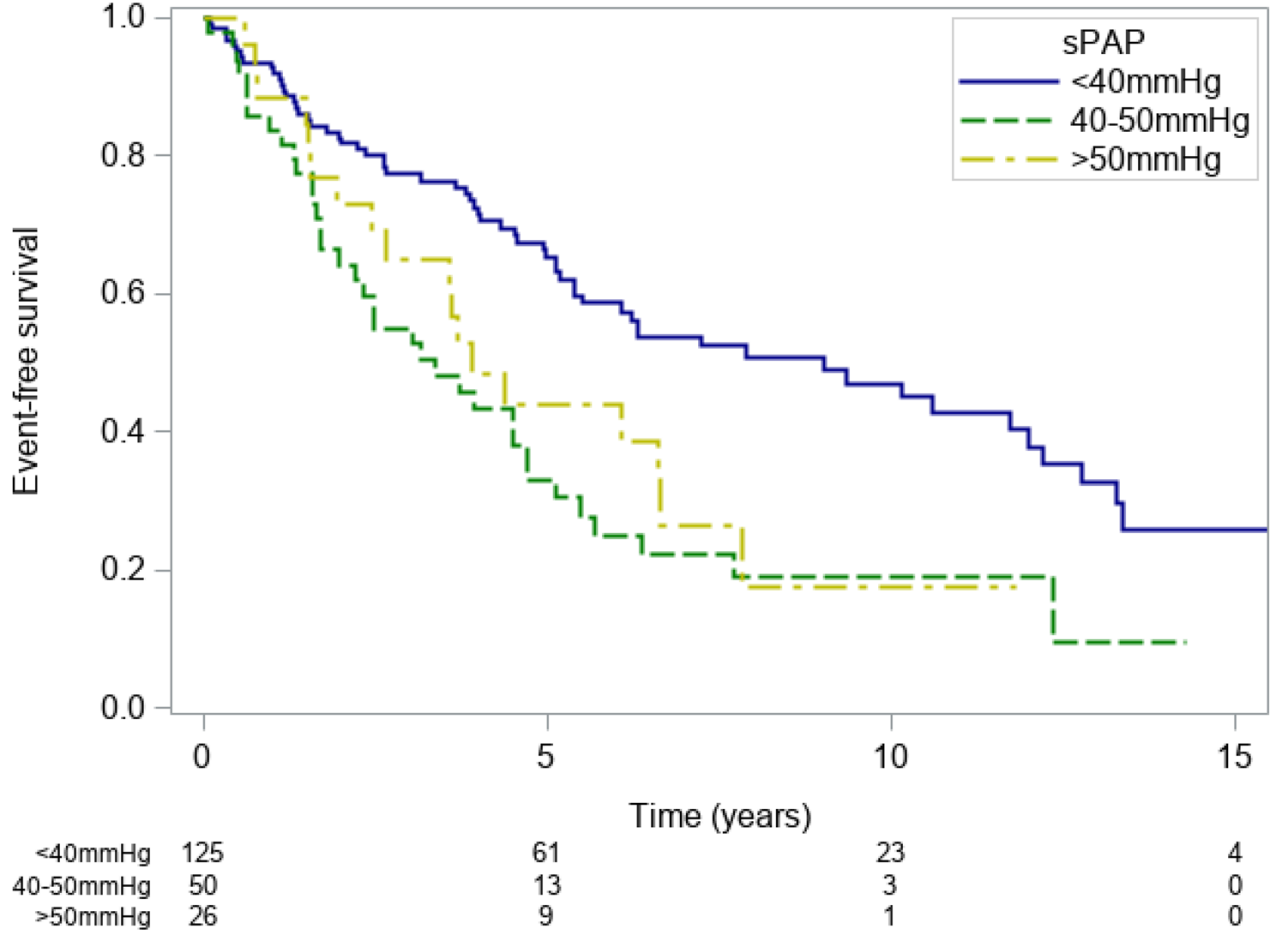
Kaplan–Meier event-free survival estimates for patients with sPAP < 40 mmHg (blue line), 40–50 mmHg (green line) and > 50 mmHg (yellow line). *sPAP* systolic pulmonary artery pressure.

Furthermore, systolic pulmonary artery pressure (sPAP) was associated with event-free survival. The respective event-free survival rates were:

- 81.8% (73.6–87.6%), 58.6% (48.5–67.4%) and 47.0% (36.1–57.2%) at 2,6 and 10 years for patients with sPAP < 40 mmHg
- 64.2% (48.8–76.0%), 25.0% (13.0–38.9%) and 19.0% (8.5–32.7%) for patients with sPAP 40–50 mmHG and
- 73.1% (51.7–86.2%), 44.0% (24.2–62.1%) and 17.6% (3.8–39.6%) for patients with sPAP > 50 mmHg (p < 0.001; PEV = 5.8%, Fig. 3).

In a multivariable analysis that encompassed age at baseline, previous history of AF, left ventricular end systolic diameter (LVSD), LA diameter (as continuous variable), sPAP > 50 mmHg and year of inclusion, LA diameter was the strongest independent echocardiographic predictor of event-free survival (adjusted HR = 1.039, p < 0.001), adding 2.5% points of explained variation to the 16.1% explained by the remaining predictors (partial PEV = 2.5%, Table 2).

**Table 2:** Predictors of event-free survival.

Parameter	Unadjusted HR (95% CI)	Unadjusted P-value	Marginal PEV %	Adjusted HR (95% CI)	Adjusted P value	Partial PEV %
LVSD (mm)^a^	1.03 (0.99; 10.6)	0.001	2.4	1.04 (1.01; 1.07)	0.028	2.1
Age (years)	1.01 (1.00; 1.02)	0.012	2.2	1.01 (0.99; 1.02)	0.413	0.2
Left atrial diameter (mm)	1.04 (1.03; 1.06)	< 0.001	5.4	1.04 (1.02; 1.06)	< 0.001	2.5
sPAP > 50 mmHg	1.58 (0.96; 2.58)	0.072	1.0	0.76 (0.40; 1.43)	0.392	0.3
Previous episode of AF	2.29 (1.53; 3.43)	< 0.001	3.0	1.44 (0.86; 2.41)	0.167	0.3
Year of inclusion (years)	1.08 (1.04; 1.12)	< 0.001	11.2	1.10 (1.05; 1.15)	< 0.001	11.5

*HR* hazard ratio, *PEV* proportion of explained variation.

^a^LVSD modelled with linear and quadratic term, HR evaluated at LVSD = 32.4 (mean).

### Surgery and surgical outcomes

During follow-up, 147 patients underwent surgery and mitral repair was performed in 127 patients (86%). There were no deaths in the perioperative (within 30 days of surgery) or early postoperative (1 to 3 months after surgery) phase but 1 patient died 94 days after mechanical mitral valve replacement and tricuspid repair surgery while still in the intensive care unit, following a postoperative phase complicated by bacterial and fungal pneumonia, as well as gastrointestinal bleeding and pericardial tamponade.

### Overall survival

Overall survival including perioperative and late deaths after surgery was 99.6% (95% CI: 98.9–100%) at 2 years, 94.6% (95% CI: 91.7–97.6%) at 6 years, 85.6% (95% CI: 80.3–91.2%) at 10 years, and 74.5% (95% CI: 66.6–83.4%) at 15 years. As reported previously^4^, survival of the study population was comparable with the expected cumulative survival.

### Predictors of overall survival

**Figure 4:**
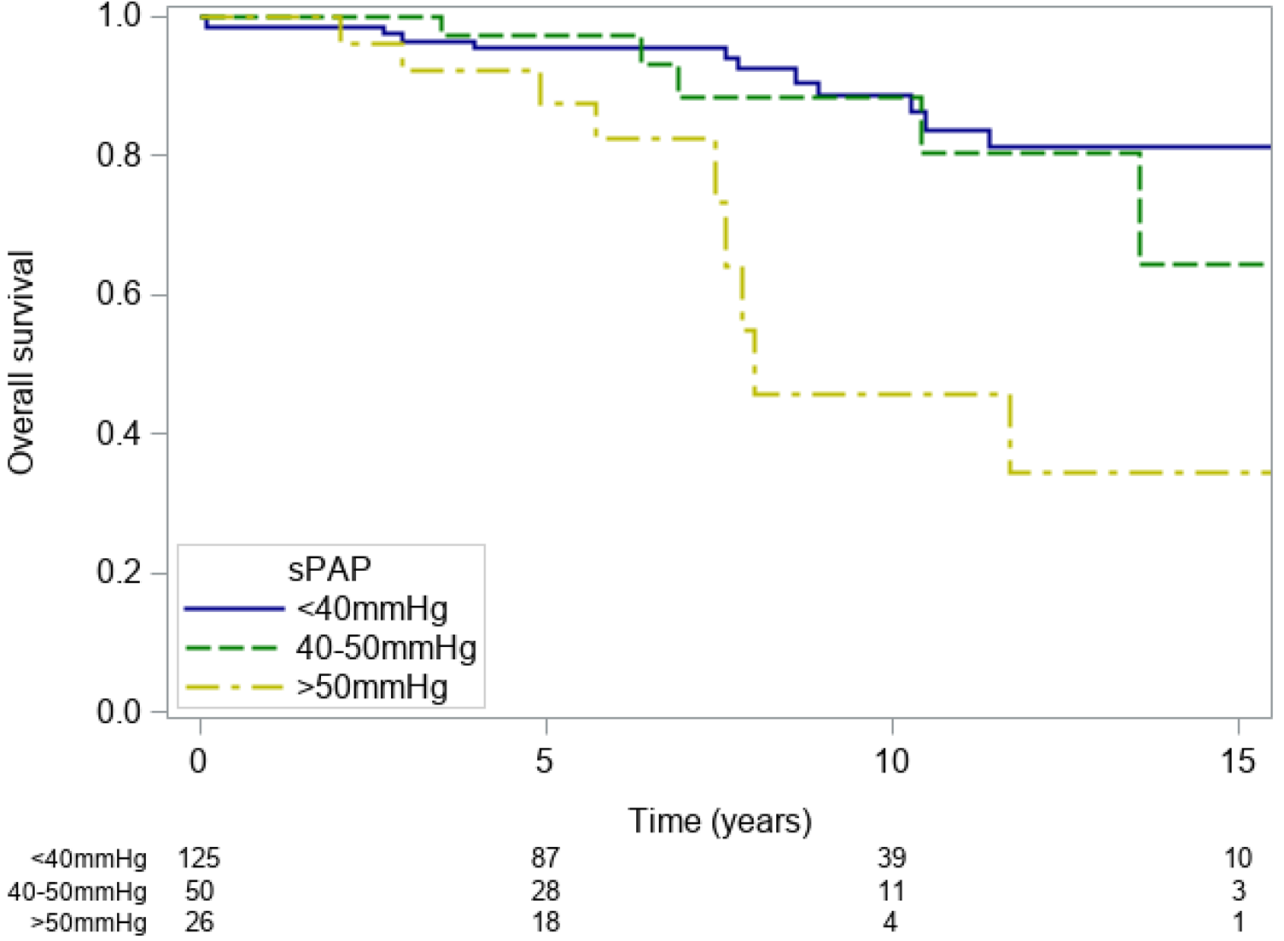
Kaplan–Meier overall survival estimates for patients with sPAP < 40 mmHg (blue line), 40–50 mmHg (green line) and > 50 mmHg (yellow line). *sPAP* systolic pulmonary artery pressure.

In the univariable analysis, sPAP was associated with overall survival. The respective survival rates were:

- 98.4% (93.8–99.6%), 95.6% (89.6–98.1%), 88.7% (78.5–94.2%) and 81.2% (67.7–89.5%) at 2,6, 10 and 15 years for patients with sPAP < 40 mmHg,
- 100%, 97.3% (82.3–99.6%), 88.4% (67.4–96.2%) and 64.3% (25.6–86.7%) for patients with sPAP 40–50 mmHg and
- 100%, 82.4% (59.2–93.1%), 45.8% (18.0–70.1%) and 34.3% (9.7–61.3%) for patients with sPAP > 50 mmHg (p < 0.001 Fig. 4).

When adjusting for age and year of inclusion however, the hazard ratio of PH for survival decreased from HR = 4.28 (p < 0.001) to an age-adjusted HR = 1.87 which was no longer significant (p = 0.126).

Furthermore, in the univariable analysis, a LA diameter > 70 mm was associated with overall survival. The respective overall survival rates were

- 98.8% (96.4–99.6%), 94.4% (90.2–96.8%), 86.1% (79.4–90.7%) and 75.3% (65.3–82.7%) at 2,6, 10 and 15 years for patients with an LA diameter ≤ 70 mm and
- 100%, 88.8% (62.1–97.1%), 72.4% (40.6–89.0%) and 60.3% (26.5–82.5%) for patients with an LA diameter > 70 mm (p = 0.033).

Due to the low number of events (31) adjustment was only performed with respect to the strongest predictor, age (PEV = 26.5%) and year of inclusion. The hazard of overall death is increased by 4.5% per additional mm in LA diameter (HR = 1.045, 95% CI: 1.01 to 1.08, p = 0.009).

Adjusting for age and year of inclusion, this effect decreases to 3.4% per additional mm (adjusted HR = 1.034, 95% CI: 1.00 to 1.07, p = 0.057; partial PEV = 3.8%).

### LA diameter vs. LA volume

In an additional analysis of the 178 patients that had been included after beginning of 2005, when LA volumes were routinely measured, we analyzed the prognostic impact of LA volumes as compared to LA diameters.

**Figure 5:**
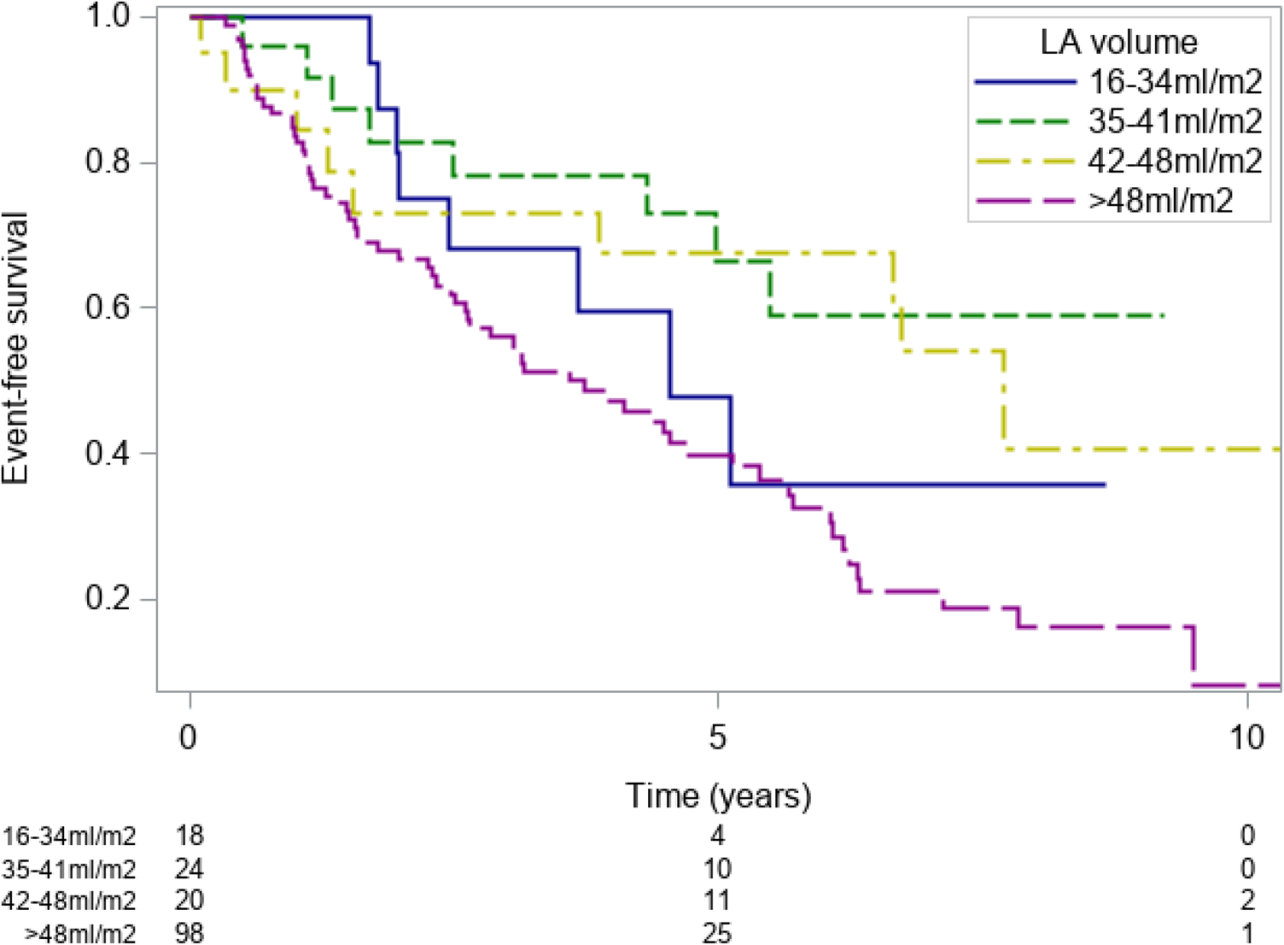
Kaplan–Meier event-free survival estimates patients included after beginning of 2005 with an indexed LA volume of 16-34 ml/m^2^ (blue line), an indexed LA volume of 35-41 ml/m^2^ (green line), an indexed LA volume of 42-48 ml/m^2^ (yellow line) and an indexed LA volume of > 48 ml/m^2^ (purple line).

- When stratifying the patients according to the proposed thresholds of indexed LA volumes^10^, event-free survival rates for patients with an indexed LA volume of 16–34 ml/m^2^ were 75.0% (46.3–89.7%) at 2 years and 35.7% (9.9–63.4%) at 6 years;
- Patients with an indexed LA volume of 35–41 ml/m^2^ had event-free survival rates of 82.9% (60.5–93.2%) at 2 years, and 59.0% (33.0–77.8%) at 6 years;
- Patients with an indexed LA volume of 42–48 ml/m^2^ had event-free survival rates of 73.1% (46.8–87.9%) at 2 years, 67.5% (41.3–84.0%) at 6 years and 41.0% (13.5–66.5%) at 10 years;
- Patients with an indexed LA volume > 48 ml/m^2^ had event-free survival rates of 66.6% (56.2–75.1%) at 2 years, 32.4% (21.9–43.4%) at 6 years and 8.0% (1.0–25.2%) at 10 years (p = 0.004, Fig. 5).

The seemingly non-linear effect most likely reflects the relatively low number of events in the low LA volume groups, and was not statistically significant.

**Figure 6:**
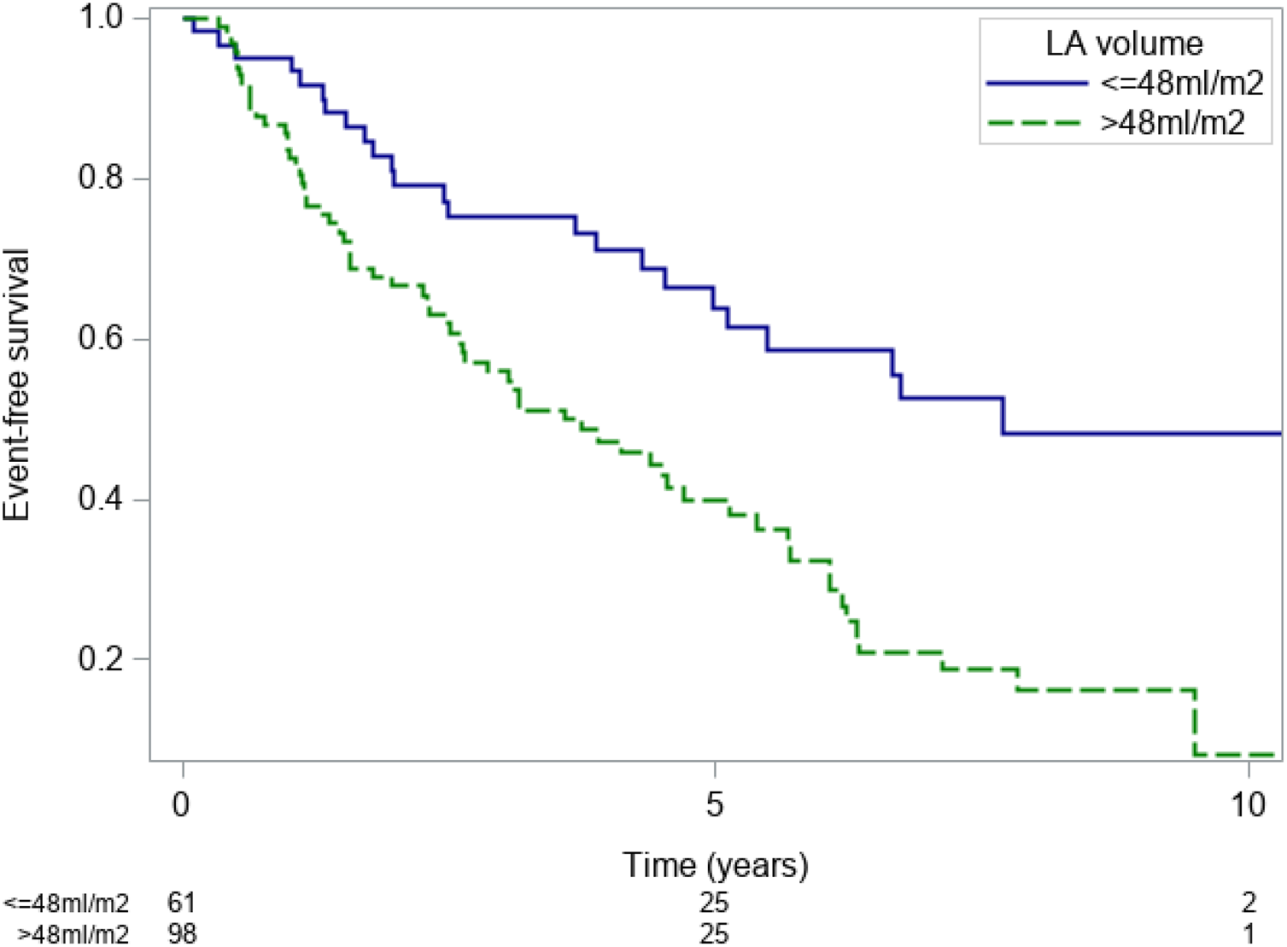
Kaplan–Meier event-free survival estimates patients included after beginning of 2005 with an indexed LA volume of ≤ 48 ml/m^2^ (blue line) and > 48 ml/m^2^ (green line).

When stratifying the patients according to an indexed LA volume of > 48 ml/m^2^, Kaplan–Meier event-free survival curves separated significantly with respective event-free survival rates of 67% (56–75%), 32% (22–43%) and 8% (1–25%) at 2, 6 and 10 years respectively for patients with an indexed LA volume of > 48 ml/m^2^ as compared to 79% (66–88%), 59% (43–71%) and 48% (32–63%) at 2, 6 and 10 years respectively for patients with an indexed LA volume of ≤ 48 ml/m^2^ (p < 0.001, Fig. 6).

When stratifying according to LA diameters, event-free survival rates were

- 81.0% (60.1–91.6%) at 2 years and 56.6% (31.2–75.7%) at 6 years for patients with an LA diameter ≤ 50 mm;
- 76.5% (66.2–84.0%) at 2 years, 51.0% (39.0–61.8%) at 6 years and 24.9% (7.4–47.4%) at 10 years for patients with an LA diameter of 51–60 mm;
- 63.4% (46.1–76.5%) at 2 years, 31.0% (15.8–47.3%) at 6 years and 16.6% (5.1–33.7%) at 10 years for patients with an LA diameter of 61–70 mm.
- 47.1% (23.0–68.0%) at 2 years and 14.7% (2.6–36.4%) at 6 years for patients with an LA diameter > 70 mm (p < 0.001).

Kaplan Meier curves for the subcohort starting from 2005 are similar to Fig. 1, albeit slightly lower.

Both LA diameter and indexed LA volume were significant predictors of event-free survival in both unadjusted (LA diameter: HR = 1.04, p < 0.001; LA volume: HR = 1.02, p < 0.001) and adjusted analyses (LA diameter: HR = 1.04, p = 0.011; LA volume: HR = 1.02, p = 0.001), respectively.

In a multivariable analysis encompassing age at baseline, previous history of AF, LVSD, sPAP > 50 mmHg and year of inclusion, marginal PEVs for LA diameter and indexed LA volume were 3.9% and 6.7%, respectively (p = 0.302). While the other variables (age at baseline, previous history of AF, LVSD, sPAP > 50 mmHg and year of inclusion) have a cumulative PEV of 11.5%,. the partial PEVs (representing the supplementary PEV of any added variable) were 1.9% and 3.4% for LA diameter and LA volume, respectively (p = 0.126).

With regards to predictors of overall survival in this particular subanalysis, only 13 events occurred, warranting only unadjusted analyses. These revealed no statistically significant predictive effect of LA diameter (HR = 1.03, p = 0.378, marginal PEV = 2.8%) or LA volume (HR = 1.01, p = 0.507, marginal PEV = 4.9%); (p = 0.420).

## Discussion

### Prognostic impact of LA size

LA size has been denominated the “hemoglobin A1c” of the heart, reflecting both elevated filling pressures due to LV systolic and diastolic dysfunction and the effects of chronic MR. LA enlargement is thus a criterion of plausibility for the diagnosis of severe MR^7,8^. Traditionally, LA enlargement has been viewed as a compensatory mechanism to offset elevated filling pressures, however LA remodeling in the setting of MR has also been demonstrated to confer deleterious effects, such as compromise in atrial booster-pump function^11^ and increased LA size itself might lead to neurohumoral responses that could aggravate remodeling and lead to a worse prognosis^12^.

Thus, the size of the LA, being the chamber affected primarily by the presence of MR might yield prognostic value in these patients. LA volume index was demonstrated to be predictive of long-term survival in patients with various degrees of primary MR that were not systematically followed in a dedicated HVC^13^. In the aforementioned study however, a cut-off of 60 ml/m^2^ was proposed, contrasting with current recommendations for chamber quantification that recommend a cutoff of 48 ml/m^2^ for the definition of severe LA dilation^10^. Most recently, data from the MIDA registry demonstrated an independent association of LA size and mortality in patients with various degrees of primary MR. LA size was quantitated and analyzed as a continuous variable and categorized at 3 levels of severity (< 40, 40 to 59, and ≥ 60 ml/m^2^), with normal size being 27 ± 7 ml/m^2^. In that study however, symptomatic patients were included, 70% of the patients did not have severe MR and therapeutic management was decided by patient’s personal physicians^14^.

The present study, to our best knowledge, is the first to show an incremental prognostic impact of LA size on event-free survival in asymptomatic patients with severe primary MR who were rigorously followed in a tertiary care HVC. In particular, left atrial diameter reliably predicted outcome across the entire spectrum from normal to severely enlarged. Over 50% of patients with a LA diameter > 70 mm required surgery after 2 years. In accordance, LA volume index, the currently recommended gold standard of LA measurement^10^, was associated with event-free survival at the established cutoff of 48 ml/m^2^. However, the small incremental prognostic value by replacing non-indexed diameters with LA volume index across the entire size spectrum was not statistically significant. LA diameter is a simple, easily obtainable and well reproducible marker of prognosis in severe asymptomatic MR, allowing for accurate risk stratification and optimized management of these patients. In particular, when adjusted for other established risk markers in severe chronic primary MR such as LVSD, PH or AF, LA diameter remained the strongest independent echocardiographic predictor of subsequent events. The significant predictive value added by the year of inclusion most likely reflects the more rigorous assessment of symptoms in individual patients as well as the consequent application of guideline recommendations since the establishment of a dedicated HVC.

### Prognostic impact of pulmonary hypertension

PH is found in up to 20% of patients with severe asymptomatic primary MR with preserved LV ejection fraction^15^ and is associated with heart failure and poor survival^16^. PH, defined by an sPAP ≥ 50 mmHg, is thus an established surgical indication in the ESC/EACTS guidelines, and in the present study was associated with lower event-free survival in asymptomatic patients. This effect also held true for a lower cut-off value of 40 mmHg that allowed for distinguishing patients that remained asymptomatic from those that rapidly developed symptoms. Importantly, in accordance with previous studies^17^, PH was associated with worse overall survival, in particular at the established cut-off of 50 mmHg but to a lesser degree even at a cut-off of 40 mmHg. This effect however, was not independent of age.

### Prognostic impact of atrial fibrillation

Since MR causes LA enlargement and remodeling, AF is a frequent complication characterizing its long-term course. While some authors regard AF’s role as a prognostic marker insignificant^18^, other studies have demonstrated an association with mortality in patients with MR that were managed conservatively^19^ and after mitral surgery^20^. Therefore, American and European guidelines on valvular heart disease consider AF to be an indication for mitral surgery^2,3^.

While patients needed to be asymptomatic and in sinus rhythm in order to be included into the present study, previous episodes of AF had been documented in 13% of the patients and were associated with poor event-free survival in the univariable analysis. In accordance, recently published data from the MIDA registry^21^ indicate that AF is a strong predictor of death in patients with severe primary MR, even after adjusting for other known risk factors and for Class I recommendations for MV surgery. In that report, surgery was significantly associated with survival, with no significant interactive effect between surgery and heart rhythm. Strikingly, perioperative mortality rate was higher in patients with pre-operative AF and landmark analysis revealed a beneficial effect of surgery when performed within 6 months of diagnosis, regardless of rhythm status.

### Timing of surgery in asymptomatic severe MR

Current AHA/ACC guidelines^3^ recommend elective mitral valve repair to be considered if the operative risk is low and the likelihood of successful repair is > 95% whereas the ESC/EACTS guidelines^2^ are more restrictive, recommending the consideration of surgery only when the LV is enlarged in the presence of either a flail leaflet or significant LA dilatation when a durable repair is likely, surgical risk is low, and the repair is performed in a heart valve center.

Predictors of outcome, defining high-risk subgroups of asymptomatic patients that might nevertheless benefit from early surgery are scarce. Regurgitation severity, as defined by effective regurgitant orifice area (EROA) has been reported to be associated with survival in asymptomatic patients^5^. In that study however, patients were not systematically followed-up in a dedicated valve clinic. In addition, the use of a single parameter to determine regurgitation severity and indicate surgery might be problematic, in particular since interobserver agreement of quantitative parameters for the grading of MR is only close to 50%^22^.

While thus, a general recommendation of early elective surgery in these patients does not seem justified, the following implications regarding the timing of surgery in patients with severe asymptomatic MR might be drawn from our findings:

1. LA size is a strong, independent predictor of outcome in patients with severe asymptomatic MR. In particular, LA diameters are easily obtainable and reliably associated with outcome across the entire spectrum of LA enlargement. The group with an LA diameter > 70 mm had markedly high event-rates and might thus be considered for early elective surgery by experienced surgeons in high-volume centers after careful evaluation. This is further supported by recent findings, indicating that after mitral surgery, excess mortality in such patients is mostly alleviated, but remains detectable for patients with severe LA enlargement (defined by a LA volume index ≥ 60 ml/m^2^ in that particular study)^14^.
2. High event-rates for asymptomatic patients with elevated sPAP and its impact on overall survival, suggest that sPAP should be an important element in the decision-making process in these patients. On the other hand, sPAP was not independent of age in the present study, thus lowering the cut-off to indicate surgery does seem not justified. In addition, bearing in mind the inherent difficulties and pitfalls in determining sPAP by echocardiography^10^ and the lesser specificity of a lower threshold, using sPAP as a sole criterion to indicate surgery in asymptomatic patients might be problematic and therefore, other signs of decreased compensatory reserve or impending symptomatic deterioration, such as LA enlargement should be present when considering a patient for early elective valve repair based on the presence of PH.
3. Even a solitary episode of AF has been shown to affect event-free survival and reportedly impacts survival^21^, thus supporting the value of MV surgery in patients with severe primary MR after the first onset of AF.

### Study limitations

Surgical indications for asymptomatic primary MR have changed during the study period, mainly owing to the addition of surgical indications in selected low-risk asymptomatic patients. None of the patients in our study however were operated on for those latter reasons.

It might be viewed as a limitation that the assessment of symptoms may be challenging and exercise testing, was not performed systematically. At the same time, a structured physician-based assessment of symptoms that included the use of exercise testing in selected patients yielded excellent long-term survival.sPAP was not measurable in 79 patients due to the absence of tricuspid regurgitation. In a separate analysis, the overall and event-free survival of these latter patients did not differ significantly from those in whom a sPAP < 40 mmHg could be estimated by echocardiography.

Quantitation of MR severity may be challenging. In the present study, an integrated approach was used to quantify MR, as suggested by current recommendations and, furthermore, the majority of the patients had flail leaflets which is highly suggestive of the presence of severe MR. It should be noted however, that it had been demonstrated recently, that in some patients, flail leaflets might not necessarly cause severe MR^23^. An integrated, multiparametric approach to quantifying MR is thus warranted.

While serum biomarkers were not routinely assessed in this patient population, no consistent threshold of serum N-terminal pro–B-type natriuretic peptide concentration has been defined yet to select patients for early surgery.

## Conclusion

In patients with asymptomatic severe MR, LA size may differentiate between patients remaining free of indications for surgery for an extensive period and those who are likely to develop such indications in the near future. Left atrial diameter is a simple and reproducible parameter, helping to identify patients who may benefit from early elective valve repair in heart valve centers of excellence.

## Data Availability

The datasets used and/or analysed during the current study are available from the corresponding author on reasonable request.
